# Modulation effects of imagery acupuncture and no-touch double-blinded placebo acupuncture, a cross-over pilot study

**DOI:** 10.1016/j.bbii.2024.100068

**Published:** 2024-07-06

**Authors:** Nobuari Takakura, Valeria Sacca, Miho Takayama, Qiao Kong, Tomohiro Tanaka, Takahiro Yamada, Konomi Imanishi, Amy Katherine Ursitti, Meixuan Zhu, Hiroyoshi Yajima, Jian Kong

**Affiliations:** a Department of Acupuncture and Moxibustion, Tokyo Ariake University of Medical and Health Sciences, 2-9-1 Ariake, Koto-ku, Tokyo 135-0063, Japan; b Department of Psychiatry, Massachusetts General Hospital, Charlestown, MA, 02129, USA

**Keywords:** Video-guided acupuncture imagery treatment, E-health, Placebo acupuncture, EEG, Double-blind placebo needle, No-touch placebo needle

## Abstract

Both imagery and acupuncture are the oldest medical practices. Recently, we have developed a new treatment modality, video-guided acupuncture imagery treatment (VGAIT), which combines acupuncture and imagery. In this crossover study, we investigated the modulation effects of video-guided acupuncture imagery treatment compared with placebo acupuncture using no-touch double-blind placebo acupuncture needles and a no-treatment resting control. Pressure pain threshold and electroencephalogram (EEG) data were collected before and after each intervention. 12 healthy participants completed the study. Results showed that pressure pain thresholds were significantly increased after VGAIT compared to the resting control condition. In addition, we found that VGAIT, but not the no-touch placebo acupuncture or the resting control, significantly increased alpha and beta band power. Our findings demonstrate the potential of VGAIT as a remote therapeutic method (e-health treatment option) for pain and the value of no-touch double-blind placebo acupuncture in acupuncture research.

## Introduction

The use of imagery to treat illness is one of the oldest medical practices (Pincus and Sheikh, 2009; Sheikh, 2003). Although still under investigation, research findings support the notion that the brain responds to imagined experience in a similar way to actual experience (Meier et al., 2012; Kosslyn et al., 2001; Ochsner et al., 2008; Ogino et al., 2007; Mochizuki et al., 2013; Christian et al., 2015; Singer et al., 2009, 2004; Lamm et al., 2011; Rutgen et al., 2015). Combining acupuncture and imagery, we have developed a new treatment modality, video-guided acupuncture imagery treatment (VGAIT). During treatment using VGAIT, the participant watched a video of acupuncture being administered on him/herself while imagining it being concurrently applied to him/her own body. We found that VGAIT can 1) produce an analgesic effect as well as brain activity changes in the anterior insula and anterior cingulate cortex (ACC), key regions involved in pain perception and modulation (Cao et al., 2019), and 2) modulate the functional connectivity of the periaqueductal grey (PAG) (Cao et al., 2021) and thalamocortical networks (Kong et al., 2023) in healthy participants. In a more recent pilot study, we found that VGAIT can produce comparable symptom relief to that obtained after one month (6 treatments) of real acupuncture intervention in chronic low back pain (cLBP) patients (Cao et al., 2020a). Thus, VGAIT may hold the potential to serve as a supplementary intervention of acupuncture, which can be applied remotely.

Acupuncture is an Eastern medical intervention with a long history that has gained traction recently due to its pain-relieving qualities (Berman et al., 2010; Vickers and Linde, 2014), cost-effectiveness (Ratcliffe et al., 2006; Ochi, 2013; Lin et al., 2011), and minimal risk of adverse side effects (Ochi, 2013; MacPherson et al., 2001; White et al., 2001; Melchart et al., 2004). Nevertheless, clinical trials evaluating the efficacy of acupuncture treatment have yielded inconsistent results (Linde et al., 2005, 2007; Melchart et al., 2005; Scharf et al., 2006; Vickers et al., 2012; Witt et al., 2005, 2006; Cherkin et al., 2009; MacPherson et al., 2013; Hinman et al., 2014) and found only modest effects that can be directly ascribed to the acupuncture treatment itself. One potential reason for these findings may be due to the fact that minimal, superficial, sham, or ‘placebo’ acupuncture (commonly used in acupuncture research) may stimulate mechanoreceptors coupled to slow-conducting afferents (Lund and Lundeberg, 2006) and thereby increase activity in the insular region (Olausson et al., 2002). These findings suggest that placebo acupuncture applied in some previous studies may alleviate the affective component of pain, so it may not be completely inert. A control condition that does not lead to such effects is needed to elucidate the efficacy of acupuncture treatment.

The double-blind, placebo-controlled clinical trial is the gold standard for showing that a treatment has a specific effect over placebo (Feinstein, 1984; Vase et al., 2015). As previously mentioned, one challenge in acupuncture research has been finding a completely inert and double-blinded placebo condition for comparison with real acupuncture and other interventions. Fortunately, investigators have developed a double-blinded acupuncture needle for acupuncture research (Takakura and Yajima, 2007). More intriguingly, a double-blinded, no-touch placebo acupuncture needle that does not make contact with the skin has been developed (Takakura et al., 2011; Takayama et al., 2015). This needle, an inert placebo based on both modern physiology and traditional acupuncture theory, may hold the potential to solve this challenge in acupuncture research.

Although still under investigation, studies suggest that both imagery and placebo work through top-down modulation, representing the power of the brain (mind) (Kong and Eshel, 2020). However, to our knowledge, no studies have compared VGAIT and placebo acupuncture directly. Such a comparison may help us explore the different brain mechanisms associated with these acupuncture-related methods and contexts, facilitate the development of new therapeutic methods (for pain management).

Recently, electroencephalogram (EEG) and magnetoencephalography (MEG) have been applied to investigate imagery (Pidgeon et al., 2016; Wilson et al., 2016; Harrison et al., 2017), acupuncture (Sakai et al., 2007), and placebo analgesia (Li et al., 2016; Tu et al., 2019; De Pascalis and Scacchia, 2019). In a previous study, we found that alpha band brain connectivity changes before and after conditioning could predict the magnitude of placebo and nocebo effects (Tu et al., 2019). EEG studies also revealed a tendency for altered alpha band power during creative visual imagery compared to baseline (Pidgeon et al., 2016). Thus, in this study, we comparatively investigated the analgesic effects and EEG activity changes produced by VGAIT, no-touch placebo acupuncture, and resting control.

## Experimental design and methods

### Participants

12 healthy volunteers were recruited in this crossover study. Participants were recruited by advertisement posted in Tokyo Ariake University of Medical and Health Sciences. All participants have received acupuncture in the past. This study was approved by the Ethics Committee of Tokyo Ariake University of Medical and Health Sciences (Approval no. 355, date of approval: 19 July 2021). Participant recruitment began in July 2022, and all data were obtained between July 2022 and September 2022. All participants signed the consent form before the initiation of the experiment. Compensation was not provided.

Randomization: In this crossover study, the three interventions/conditions (VGAIT, placebo acupuncture using no-touch double-blinded placebo needles, and resting control) were performed in a random order (randomization was generated by a website (http://randomization.com/). Six orders were used, each order was used twice (two subjects). 12 envelopes with the order of implementation of the three conditions were randomly numbered from 1 to 12. Participants were numbered 1–12 in order of enrollment and allotted the randomization in the envelope with the matching number. The interval between each intervention was at least 24 hours (mean ± standard deviation day of the intervals was 9.6 ± 9.6).

Blinding: The participants, the acupuncturist, and the research assistants were blinded to the fact that the acupuncture intervention was placebo acupuncture. In order to ensure that the acupuncturist was blinded, the acupuncturist randomly took one needle from a bag containing 12 double-blind needles. The acupuncturist was informed that there were both real and placebo needles in the bag; however, only no-touch placebo needles were included. The participants did not know there would be placebo acupuncture. They were only informed that they would receive acupuncture. The research assistants who collected pressure pain threshold measurements, EEG data, and questionnaires were blinded to the condition that the participant received.

## Research procedure

### EEG measurement

EEG data were collected for 6 minutes before, during, and after treatment. EEG measurements were performed in a shielded room with radio frequency shielding. Each participant sat in a chair with a backrest and armrests. 19 electrodes were attached according to the international 10–20 system. Reference electrodes were attached to the left and right earlobes, and the electroactivity in the brain from each electrode was derived. Two electrooculograms were recorded with electrodes placed at the infra- and supra- orbital areas on the outer left eye. EEG and electrooculogram were measured with an EEG recording system (Neurofax EEG-1200, Nihon Kohden, Tokyo, Japan). EEG was sampled at 1000 Hz through a 0.1–100 Hz bandpass filter. Impedance was kept below 5 kΩ during EEG data collection. The participant was instructed to gaze at a black cross mark on a white background on the monitor during EEG measurements before and after the interventions and during the resting control. During VGAIT, the participant was instructed to focus on a screen showing a video of him/herself receiving acupuncture.

### Pain measurement

Pressure pain threshold: Pressure pain thresholds were measured before and after the EEG recording in each condition. The tip of the algometer probe (area: 0.5 cm^2^) was placed on the base of the thumbnail on each hand of the participant, and then the intensity of the applied pressure gradually increased at a constant speed. When the participant first felt pain from the pressure on the thumb, he/she signaled it with the opposite hand. This measurement was repeated three times on each hand. Mean values from the three trials were calculated to determine the participant’s pressure pain threshold before the intervention (maximum pressure value: 40 kilopascals). We averaged left and right pain thresholds during data analysis to avoid *p*-value correction for multiple tests.

### Psychometric measurements

#### Massachusetts General Hospital (MGH) Acupuncture Sensation Scale (MASS, Japanese version):

The MASS includes 12 descriptors of common acupuncture sensations: soreness, aching, deep pressure, heaviness, fullness/distension, tingling, numbness, sharp pain, dull pain, warmth, cold, and throbbing (Kong et al., 2007; Nishiwaki et al., 2017). Participants were asked to rate the acupuncture sensations they felt on the MASS from 0 to 10 after receiving VGAIT and no-touch placebo needle acupuncture. They were also given an opportunity to describe their perceptions in their own words.

#### Sensory visual analogue scale (S-VAS):

the scale is used to measure the intensity of sensations from 0 (no sensation) to 100 (most severe sensation, unbearable) when they received acupuncture or VIGAIT.

#### Expectations for Relief Scale (ERS):

The ERS is a 0–10 scale used to measure the expectation of post-intervention pain relief. Participants completed this assessment before VGAIT and no-touch placebo needle acupuncture.

## Interventions

### Video preparation

**Fig. 1.**
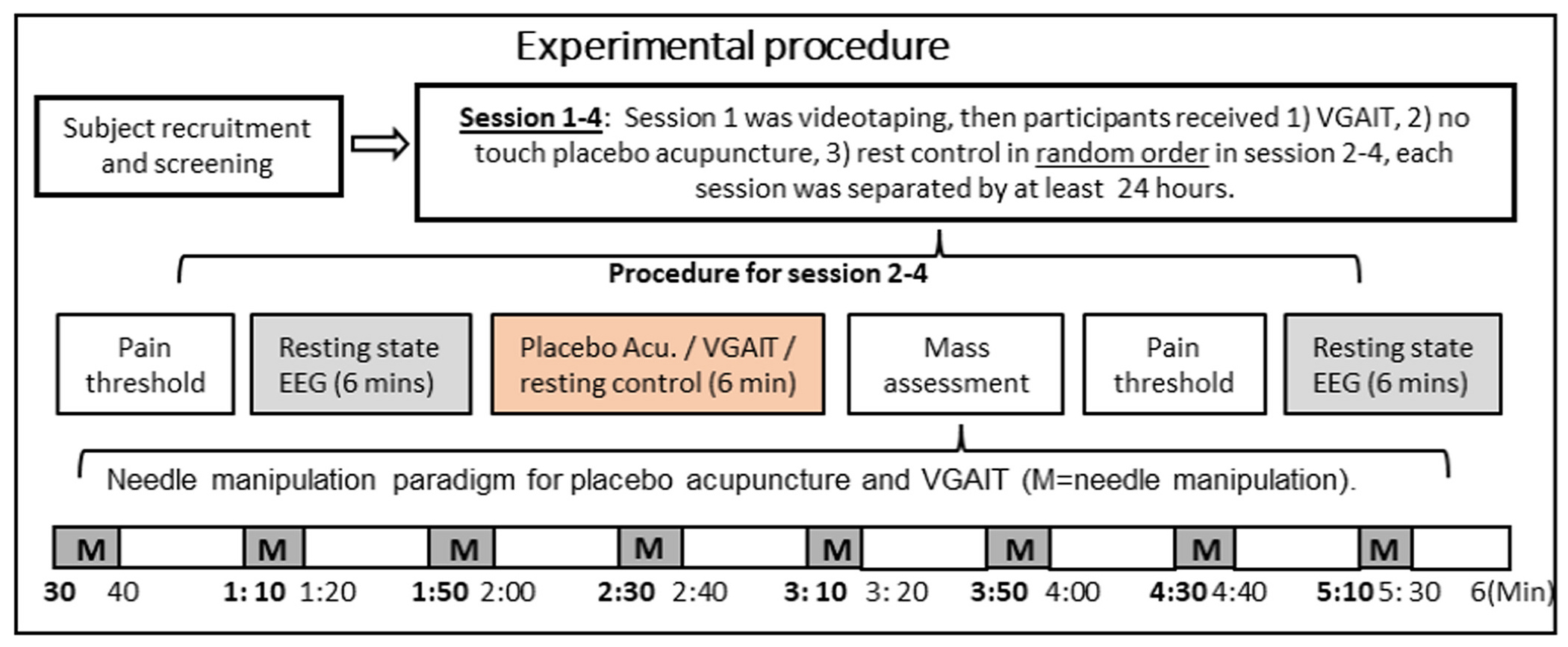
Experimental design: healthy participants were recruited and received VGAIT, no-touch double-blind placebo acupuncture, and resting control in random order.

In this study, the video was taped on each participant in the lab using a classic real acupuncture needle at left LI4 at least 24 hours before the first intervention session. A video camera was placed in front of the participant, who was seated in a chair with a backrest and armrests, and the video was recorded on the left hand. The participant adjusted his/her hand position on the armrest so that the skin surface of the left LI4 was parallel to the floor. Then, the acupuncturist disinfected the skin around the left LI4, and the video recording began. The acupuncturist placed a 40 mm (No.18) stainless steel needle (Seirin Corporation, Japan) covered with a guide tube on the left LI4. The needle was inserted about 10 mm with the tapping and alternate rotating technique. The acupuncturist performed a total of 8 sets of 10 rotations of the needle at 1 Hz for 10 seconds each at the following time points: 30 seconds, 1 minute 10 seconds, 1 minute 50 seconds, 2 minutes 30 seconds, 3 minutes 10 seconds, 3 minutes 50 seconds, 4 minutes 30 seconds, and 5 minutes 10 seconds (Fig. 1). The acupuncturist then removed the needle 6 minutes after the needle was inserted, following the cues from the timekeeper.

### Video-guided acupuncture imagery treatment (VGAIT)

At the beginning of the VGAIT, participants were given the following instructions: “You will watch the video of a hand receiving acupuncture for about 6 minutes. Please imagine that you are receiving acupuncture in your left hand as vividly as you can. Even though you are only watching the acupuncture being applied, you may feel tingling, throbbing, heaviness or other acupuncture sensations in your left hand. It has been found that imagining you are receiving acupuncture or feeling acupuncture sensations while watching the video can bring you a more effective treatment, so please continue to focus on the idea that you are receiving acupuncture and try to feel the acupuncture sensations as clearly as possible. After the treatment, we will ask you about the sensations experienced.

#### Placebo acupuncture using no-touch double-blind placebo needle:

The intervention was applied by an acupuncturist blinded to the treatment mode using a double-blind no-touch placebo acupuncture needle. The participants were given the following instructions: “You will receive acupuncture for 6 minutes. Please pay attention to the manipulation of the needle. You should be able to feel tingling, throbbing, heaviness, pain or other acupuncture sensations in your left hand. It has been found that being aware of receiving acupuncture or feeling the acupuncture sensations can bring you a more effective treatment, so please continue to focus on receiving acupuncture and try to feel the acupuncture sensations as clearly as possible. After the treatment, we will ask you about the acupuncture sensations”. The treatment also lasted 6 minutes, during which the acupuncturist performed a total of 8 sets of 10 rotations of the needle at 1 Hz, identical to the VGAIT procedure.

#### Resting control:

In this session, participants followed a procedure identical to that used for VGAIT, except that they looked at a white cross with a black background instead of acupuncture videos.

## Biostatistical analysis

Data analyses were applied using JASP (version 0.17, https://jasp-stats.org/). Due to a small sample size, we performed a non-parametric paired samples test (Wilcoxon signed-rank test) for within-group comparisons to explore the modulation effects of the three conditions (VGAIT, placebo acupuncture, and resting control) on pressure pain threshold and EEG power. The Friedman test (a non-parametric alternative to the Repeated Measures ANOVA) on pre- and post- intervention measures was applied to identify differences among the three conditions. We also explored the association of acupuncture sensations, analgesia effects (pre- and post-intervention pain threshold changes), and EEG power using Kendall’s tau correlation (a non-parametric measure of relationships between columns of ranked data).

### EEG data analysis

EEG data were preprocessed using the EEGlab toolbox (https://sccn.ucsd.edu/eeglab/index.php). In a method similar to that used in a previous study (Shao et al., 2012), EEG tracks were first filtered using a band-pass filter between 0.5 and 30 Hz. Independent Component Analysis (ICA) was then performed to remove eye-blinking and eye-movement artifacts. Electrodes were then re-referenced to the average montage (Jensen et al., 2013).

The EEG spectrum was calculated for each channel using the Welch method (window length: 512 and FFT length: 1024). The absolute power density was computed for alpha (8–13 Hz) and beta (15–30 Hz) bands for each channel. We then averaged F3, F4, F7, F8, and Fz to represent the EEG spectrum for the frontal cortex; P3, P4, and Pz for the parietal cortex; C3, C4, and Cz for the central cortex; T3, T4, T5, and T6 for the temporal cortex; and O1, O2, and Oz for the occipital cortex. To assess any statistical differences in the alpha and beta bands between pre- and post-treatment within each group, a series of non-parametric paired samples tests (Wilcoxon signed-rank test) were performed. The Friedman test was applied on pre- and post- treatment measures to identify differences among the three conditions.

## Results

12 participants completed the study (6 females; age 21.3 ± 0.3, mean ± SE) and were included in the final data analysis.

Pain threshold, MASS, and their association

**Fig. 2.**
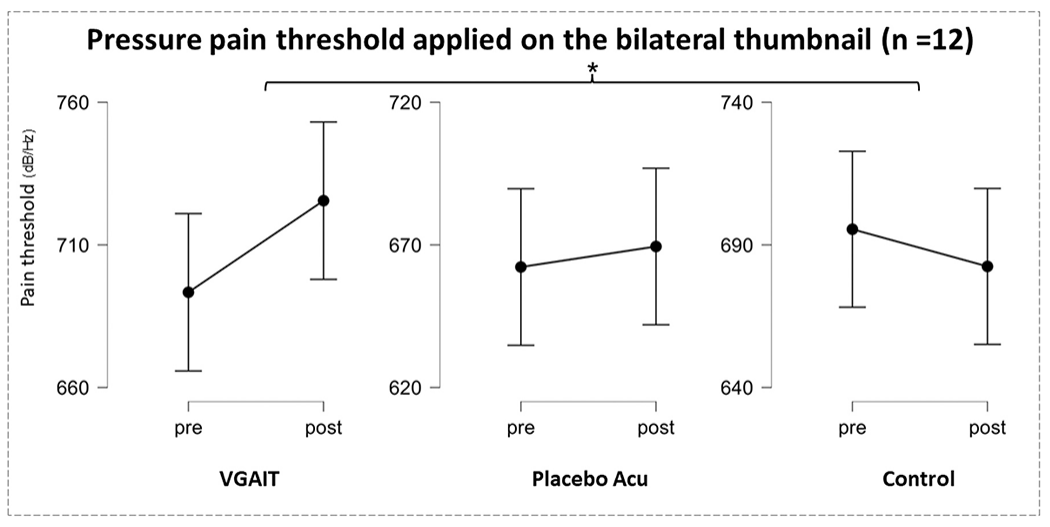
Pressure pain threshold changes before and after interventions and resting control.

Average pain thresholds before and after VGAIT, placebo acupuncture, and resting control are shown in Table 1. There was no significant difference in average pressure pain thresholds of both hands at baseline (pre-intervention) across the three conditions (Friedman test, *p* = 0.06). Within-group comparison using Wilcoxon signed-rank test showed no significant difference in average pressure pain thresholds between pre- and post- intervention in all three conditions (VGAIT, *p* = 0.11; placebo, *p* = 0.79; control, *p* = 0.38) although the three-condition showed different trends (Fig. 2).

The Friedman test on pre- and post- treatment measurements across the three conditions showed a significant difference (*p* = 0.05) in average pressure pain threshold changes among the three. Conover’s pairwise post hoc comparisons showed that the average pressure pain threshold changes of the VGAIT group were significantly greater than those of the control group (*p* = 0.02). There was no significant difference between VGAIT and placebo acupuncture groups (*p* = 0.23), nor between placebo acupuncture and control groups (*p* = 0.23).

The average MASS and ERS scores are shown in Table 1. There was no significant difference in MASS and ERS scores between VGAIT and placebo acupuncture groups (Wilcoxon signed-rank test, *p* = 0.75).

For the double-blind placebo acupuncture process, the acupuncturist reported feeling no-touch for six participants, penetrating for five participants, and unknown for one participant.

There was no significant association between MASS scores and pain threshold changes using Kendall’s tau correlation in VGAIT (*p* = 0.59) and placebo acupuncture (*p* = 0.64) conditions.

### EEG results

#### Alpha band power results

The average alpha band power before and after VGAIT, placebo acupuncture, and resting control in different brain areas is shown in Table 2. The Wilcoxon signed-rank test showed that after VGAIT, average alpha band power increased significantly in central regions (*p* = 0.03), frontal regions (*p* < 0.001), occipital regions (*p* = 0.001), parietal regions (*p* < 0.001), and temporal regions (*p* < 0.001). No significant results were observed in placebo acupuncture and control conditions.

A Friedman test on pre- and post-differences of alpha band power in the parietal region showed significant results among three conditions (*p* = 0.05). Conover’s pairwise post hoc comparisons showed that the average alpha band power changes in parietal regions of the VGAIT group were significantly greater than those of the placebo acupuncture group (*p* = 0.02). There was no significant difference between VGAIT and control conditions (*p* = 0.23), nor between placebo acupuncture and control conditions (*p* = 0.23). There were no significant results in other brain regions (*p-values* ranged from 0.34 to 0.78).

#### Beta band power results

The average beta band power before and after VGAIT, placebo acupuncture, and resting control in different brain areas is shown in Table 3. The Wilcoxon signed-rank test showed that after VGAIT, average beta band power increased significantly in central regions (*p* = 0.03), frontal regions (*p* < 0.03), occipital regions (*p* < 0.01), and parietal regions (*p* = 0.02). No significant result was found in temporal regions (*p* = 0.57). No significant results were observed in the placebo acupuncture and control conditions.

There were no significant results in pre- and post-treatment differences of beta band power among three conditions in all brain regions using the Friedman test (*p* values ranged from 0.56 to 0.72).

We also explored the association between the alpha band and beta band increase for the three conditions using Kendall’s tau correlation. For all three conditions, there were no significant correlations between the alpha and beta band power increase in all brain regions. The *p* values ranged from 0.12 to 1.00 in the VGAIT condition, 0.05–0.84 in the placebo acupuncture condition, and 0.19–0.31 in the control condition.

We further explored the association between the EEG alpha/beta band power and pain threshold changes using Kendall’s tau correlation (non-parametric). The correlation between alpha band power and pain threshold changes was not found to be significant after *p-value* correction (Bonferroni corrected *p-value* = 0.05/15 = 0.003) for all three conditions. The *p* values ranged from 0.20 to 0.84 in the VGAIT condition, 0.03–0.20 in the placebo acupuncture condition, and 0.03–0.11 in the control condition.

No significant finding was observed for the correlation between beta band power and pain threshold changes after the *p-value* correction for all three conditions. The *p* values ranged from 0.12 to 0.95 in the VGAIT group, 0.009–0.55 in the placebo acupuncture group, and 0.25–0.46 in the resting control group.

## Discussion

In this study, we investigated the modulation effects of VGAIT, placebo acupuncture using no-touch double-blind placebo acupuncture needles, and resting control. We found that VGAIT can significantly increase pressure pain thresholds (analgesic effects) compared to control condition and widespread alpha and beta band power after intervention. These findings demonstrate the potential of VGAIT as an adjunctive pain intervention.

Literature (Xie et al., 2020) has suggested that during visual mental imagery, humans conjure up a vivid internal experience from memory that stands in for the perception of the stimulus. Thus, the use of imagery as a tool has been linked to many cognitive processes, and imagery plays both symptomatic and mechanistic roles in neurological and mental disorders and treatments (Pearson, 2019).

Visually imagined experiences subjectively mimic perceived experiences. This idea suggests that imagery and perception share common neural mechanisms. In this study, we found that VGAIT can significantly increase pressure pain thresholds. This finding was consistent with that in our previous study (Cao et al., 2019, 2021, 2020b), in which we found a significant increase in pressure pain thresholds in subjects who received VGAIT compared to those who received sham VGAIT. It is worth noting that all participants in our previous study watched the acupuncture needle stimulation on their own bodies for about 20 minutes instead of 6 minutes. The results from the current study replicate and extend our previous findings and thus further endorse the potential of VGAIT as a pain management method and its implementation as a remote adjunctive therapy.

In this study, we found increased widespread alpha band power after VGAIT, but not after placebo acupuncture or the resting control. Alpha oscillations are the dominant rhythmic activity in the awake resting human brain, playing key roles in inhibitory modulation of neuronal excitability and long-range interregional communication (Sadaghiani and Kleinschmidt, 2016). Literature suggests that alpha activity may play an important role in downregulating the sensory cortex by serving the function of sensory filtering, gating, and suppression (Foxe and Snyder, 2011).

Our findings are consistent with those from previous studies suggesting that alpha activity plays an important role in imagery (Xie et al., 2020; Bartsch et al., 2015; Schaefer et al., 2011; Brinkman et al., 2014). For instance, in a previous study (Xie et al., 2020), investigators compared the oscillatory time courses of mental imagery and perception of objects. They found that representations shared between imagery and perception emerged specifically in the alpha frequency band, further suggesting that alpha oscillations may be a cortical signature of representations shared between visual mental imagery and perception.

Literature (Pearson, 2019) has suggested that visual imagery involves a network of brain areas from the frontal cortex to the parietal and temporal cortices can function much like a weak version of afferent perception. In a previous study (Dijkstra et al., 2017), investigators found that a network of areas, including both the occipital and temporal early and late visual areas, the precuneus, the right parietal cortex, and the medial frontal cortex, was involved in the experienced vividness of visual imagery. They also discovered that the more anterior areas seemed to be important for imagery-specific processes, whereas visual areas represented the visual features of the experience. Further, they found that the overlap between neural representations of imagery and perception, regardless of vividness, extended beyond the visual cortex to also include parietal and premotor/frontal areas. Our finding of increased widespread alpha band power after VGAIT is consistent with these findings.

We found alpha band power changes in parietal regions of the VGAIT group were significantly greater than those of the placebo acupuncture group. As the subjects did not receive actual sensory stimulation, we believe the effects may be due to top-down modulation. Considering the brief duration (about 6 minutes) of administration, it is unlikely that the changes in the alpha band are attributable to fatigue. Our previous fMRI study suggests a complex mechanism is involved in the VGAIT (Cao et al., 2019). Also, we did not assess the endogenous opioid system; therefore, we cannot confirm if the release of endogenous opioids is involved in this process.

We did not observe a significant difference between the VGAIT and rest. This may be due to two factors: 1) both VGAIT and placebo were associated with some level of attention focus (on a specific tasks), while less attention focus is involved during rest; 2) the small sample size. Future study with large sample size is needed to validate our finding. We also found that VGAIT can significantly increase beta activity. Beta rhythms are more often found in frontal or central areas. Previous studies have suggested that beta rhythms are involved in multiple functions such as coordination among multiple representations in the cortex, signaling of decision-making, and focusing action-selection network functions (Kropotov, 2016). The modulation effects of VGAIT on beta activity indicate that VGAIT may hold the potential to modulate these functions.

In the current study, we found that there were no significant differences in pain thresholds or EEG alpha and beta band changes after the placebo acupuncture or resting control. This result suggests that no-touch double-blinded placebo acupuncture needles did not alter brain activity or produce placebo analgesia; thus, the device may hold potential as an inert placebo control for acupuncture studies.

During the study, we neither explained the involvement of the placebo needle to the participants before treatment nor asked them to guess the authenticity of the needles. We speculated that if we asked participants to guess whether the needle was inserted into the acupoint or not, they might have attempted to guess the type of treatment received based on experienced cues (Takakura et al., 2008). This process might have elicited unexpected brain activities that could have interfered with EEG data collection.

There are several limitations in this study. First, the sample size is relatively small. Future studies with larger sample sizes are needed to further replicate the findings. Second, the crossover design may be associated with carryover effects that may confound the results. To overcome potential limitations, we have attempted to compare pre- and post- differences across different conditions, which should be able to control potential carryover effects from previous interventions.

In summary, we found that compared to the resting control, VGAIT can significantly increase pressure pain thresholds compared to resting control condition. VGAIT, but not the no-touch double-blind placebo acupuncture or resting control, can significantly increase widespread alpha and beta band power. The findings demonstrate the potential of VGAIT as a remote therapeutic method for pain. The lack of pain threshold and EEG alpha and beta power changes after no-touch double-blind placebo acupuncture suggest that the device may be a valuable placebo control for acupuncture research.

## Figures and Tables

**Table 1:** Assessments for all participants across the three conditions.

Group	VGAIT	Placebo acupuncture	Resting control
PPT (pre - intervention)	693 ± 242	662 ± 271	695 ± 274
PPT (post - intervention)	726 ± 256	669 ± 225	682 ± 266
PPT (difference (post - pre))	32 ± 61	7 ± 61	−13 ± 61
MASS score	2.1 ± 6.9	0.9 ± 1.6	-
Sensory VAS score	8.9 ± 18.2	2.6 ± 5.5	-
ERS score	3.5 ± 3.1	2.6 ± 2.2	-

Data are presented as mean ± SD. PPT: averaged pressure pain threshold (mmHg); VGAIT: video-guided acupuncture imagery treatment; STAI: State-Trait Anxiety Inventory; ERS: Expectations for Relief Scale.

**Table 2:** The alpha band power (dB/Hz, mean ± SD) before and after interventions / conditions.

	VGAIT ^+^		Placebo Acupuncture ^+^		Control	

*Brain Areas*	*Pre*	*Post*	*Pre*	*Post*	*Pre*	*Post*
Central	1.12 ± 0.91	1.24 ± 0.99*	1.20 ± 0.83	1.24 ± 0.78	1.30 ± 0.78	1.32 ± 0.74
Frontal	0.96 ± 0.56	1.17 ± 0.63*	0.94 ± 0.44	1.04 ± 0.44	1.13 ± 0.58	1.17 ± 0.53
Occipital	1.99 ± 2.04	2.55 ± 2.14*	1.77 ± 1.19	2.15 ± 1.39	2.25 ± 1.53	2.35 ± 1.49
Parietal	1.33 ± 1.04	1.76 ± 1.30*	1.49 ± 1.20	1.52 ± 0.95	1.71 ± 1.43	1.66 ± 1.27
Temporal	1.23 ± 0.78	1.51 ± 0.80*	1.15 ± 0.73	1.24 ± 0.69	1.28 ± 0.81	1.36 ± 0.76

*: significant differences (*p* < 0.05) between the within condition comparison (pre vs post).

+: significant differences between the VGAIT and Placebo acupuncture changes in parietal region (pre minus post-intervention) (*p* < 0.05).

**Table 3:** The beta band power (dB/Hz, mean ± SD) before and after interventions / conditions.

	VGAIT		Placebo Acupuncture		Control	
			
*Brain Areas*	*Pre*	*Post*	*Pre*	*Post*	*Pre*	*Post*
Central	0.26 ± 0.11	0.30 ± 0.13*	0.29 ± 0.11	0.31 ± 0.15	0.29 ± 0.12	0.35 ± 0.19
Frontal	0.40 ± 0.23	0.50 ± 0.31*	0.38 ± 0.24	0.46 ± 0.33	0.48 ± 0.45	0.54 ± 0.36
Occipital	0.29 ± 0.10	0.32 ± 0.11*	0.30 ± 0.10	0.33 ± 0.10	0.34 ± 0.15	0.34 ± 0.11
Parietal	0.23 ± 0.09	0.26 ± 0.10*	0.24 ± 0.08	0.25 ± 0.09	0.25 ± 0.13	0.27 ± 0.11
Temporal	0.47 ± 0.24	0.50 ± 0.31	0.37 ± 0.15	0.52 ± 0.57	0.49 ± 0.36	0.65 ± 0.58

*: significant differences (*p* < 0.05) between the within condition comparison (pre vs post).
